# Efficacy and safety of guselkumab in patients with active psoriatic arthritis who had inadequate efficacy and/or intolerance to one prior tumor necrosis factor inhibitor: study protocol for SOLSTICE, a phase 3B, multicenter, randomized, double-blind, placebo-controlled study

**DOI:** 10.1186/s41927-024-00386-7

**Published:** 2024-05-21

**Authors:** Alexis Ogdie, Joseph F. Merola, Philip J. Mease, Christopher T. Ritchlin, Jose U. Scher, Kimberly Parnell Lafferty, Daphne Chan, Soumya D. Chakravarty, Wayne Langholff, Yanli Wang, Olivia Choi, Yevgeniy Krol, Alice B. Gottlieb

**Affiliations:** 1 University of Pennsylvania School of Medicine, Philadelphia, PA USA; 2 Brigham and Women’s Hospital, Harvard Medical School, Boston, MA USA; 3 Swedish Medical Center Providence St. Joseph Health and University of Washington, Seattle, WA USA; 4 University of Rochester Medical Center, Rochester, NY USA; 5 New York University School of Medicine, New York, NY USA; 6 Janssen Scientific Affairs, LLC, a Johnson & Johnson company, Horsham, PA USA; 7 Drexel University College of Medicine, Philadelphia, PA USA; 8 Janssen Research & Development, LLC, Spring House, PA USA; 9 Icahn School of Medicine at Mt Sinai, New York, NY USA

**Keywords:** Psoriatic arthritis, TNFi-IR, Randomized controlled trial, Guselkumab, IL-23p19

## Abstract

**Background:**

Tumor necrosis factor inhibitors (TNFi) are frequently chosen as the first biologic for patients with psoriatic arthritis (PsA). Given that many patients with PsA are TNFi inadequate responders (TNF-IR; either inadequate efficacy or intolerance), treatments utilizing alternative mechanisms of action are needed. In phase 3 studies, the fully human ﻿interleukin (IL)-23p19 subunit-inhibitor, guselkumab, was efficacious in patients with active PsA, including TNFi-IR. Efficacy was generally consistent between TNFi-naïve and TNFi-experienced cohorts; however, in the latter, higher response rates have been observed with the Q4W dosing regimen relative to the Q8W dosing regimen for some endpoints, suggesting the need to evaluate whether more frequent dosing may provide an incremental clinical benefit for TNFi-IR patients.

**Methods:**

The phase 3b SOLSTICE study will assess guselkumab efficacy and safety in TNFi-IR PsA patients. Eligibility criteria include a PsA diagnosis for ≥ 6 months; active disease (≥ 3 swollen, ≥ 3 tender joints, C-reactive protein ≥ 0.3 mg/dL); and inadequate efficacy with, and/or intolerance to, one prior TNFi. Participants will be randomized 1:1:1 to guselkumab Q4W or Q8W or placebo→guselkumab﻿ Q4W (at Week 24). The primary endpoint is the proportion of patients achieving ≥ 20% improvement in the American College of Rheumatology criteria (ACR20) at Week 24. Major secondary endpoints include ACR50, ACR70; an Investigator’s Global Assessment (IGA) of psoriasis score of 0/1 plus ≥ 2-grade reduction and ≥ 90% improvement in Psoriasis Area and Severity Index (both among patients with ≥ 3% body surface area affected by psoriasis and baseline IGA ≥ 2); minimal/very low disease activity; and changes from baseline in Health Assessment Questionnaire-Disability Index, the 36-item Short-Form﻿ Health Survey Physical Component Summary, and Functional Assessment of Chronic Illness Therapy-Fatigue scores. The target sample size (*N* = 450) is estimated to provide > 90% power in detecting differences between each guselkumab group and the placebo group for the primary endpoint assuming a 2-sided α = 0.05. Cochran-Mantel–Haenszel testing and analyses of covariance will﻿ be used to compare efficacy for binary and continuous endpoints, respectively.

**Discussion:**

Findings from the phase 3b SOLSTICE study, the design of which was informed by results from previously conducted phase 3 studies, is expected to provide important efficacy and safety information on guselkumab therapy in TNFi-IR patients with PsA.

**Trial registration:**

This trial was registered at ClinicalTrials.gov, NCT04936308, on 23 June 2021.

**Supplementary Information:**

The online version contains supplementary material available at 10.1186/s41927-024-00386-7.

## Background

Psoriatic arthritis (PsA) is a chronic, progressive, disease characterized by inflammation across several disease domains that can lead to peripheral joint pain, skin and nail psoriasis, enthesitis, dactylitis, and axial symptoms [1]. Delays in receiving effective treatment have been associated with the development of erosive joint disease and impairments in health-related qualify of life (HRQoL) and physical function [2]. Given the heterogeneous presentation in patients with PsA, a targeted approach is recommended for the treatment of PsA based on an individual patient’s clinical manifestations, symptom severity, and comorbidities [3, 4]. Current treatment guidelines recommend directing therapy selection to treat the currently active disease features while accounting for comorbidities in individual patients. This may include the use of conventional therapies (i.e., nonsteroidal anti-inflammatory drugs [NSAIDs] or conventional synthetic disease-modifying antirheumatic drugs [csDMARDs]) or biologic (b) DMARDS such as tumor necrosis factor inhibitors (TNFi) for patients with more severe joint disease, poor prognostic factors, or more extensive skin disease [3, 5]. While TNFi therapies can be effective in achieving low levels of disease activity in some patients with PsA, a substantial proportion are considered inadequate responders to TNFi (TNFi-IR) and experience primary or secondary nonresponse and/or intolerance to such therapies [5]. Approximately 40% of patients with PsA, receiving a TNFi, do not achieve ≥ 20% improvement in the American College of Rheumatology response criteria [6] (ACR20) and over half do not achieve minimal disease activity (MDA) [7] within 6 months of treatment initiation [5, 8]. Additionally, patients who switch to a second TNFi tend to have lower response rates for achieving even modest efficacy outcomes (e.g., ACR20) [9, 10]. Currently, there are several bDMARDs and targeted synthetic DMARDs utilizing alternative mechanisms of action (e.g., inhibiting interleukin [IL]-17A, IL-23, and Janus kinase [JAK]); however, existing treatment recommendations for patients with PsA lack clear guidance on optimal treatment strategies for those who are TNFi-IR [3, 4]. Thus, understanding the efficacy of therapies like guselkumab across disease domains in TNFi-IR patients is of great clinical importance.

IL-23 is known to be a ‘master regulator’ in psoriasis [11] and has also been implicated in the pathogenesis of PsA [12]. Guselkumab (Janssen Biotech, Inc., Horsham, PA, USA) is the first and only fully human IL-23 p19-subunit inhibitor approved to treat adults with moderate-to-severe plaque psoriasis and active PsA [13]. Previously, in the randomized, double-blind, placebo-controlled, phase 2 study [14] and the randomized, double-blind, placebo-controlled phase 3 studies, DISCOVER-1 (1 year study) [15] and DISCOVER-2 (2 years﻿) [16], guselkumab-treated patients had significantly greater improvements in the signs and symptoms of PsA through Week 24 vs placebo﻿. Additionally, robust efficacy observed with guselkumab 100 mg every 4 weeks (Q4W) or Q8W was sustained through up to 2 years across disease domains [17–19]. The DISCOVER-2 study also assessed radiographic progression and demonstrated smaller least squares mean changes in PsA-modified van der Heijde-Sharp scores from baseline to Week 24 with both guselkumab dosing regimens (Q4W and Q8W) compared with placebo, with the difference between the Q4W and the placebo groups being statistically significant [16]. Rates of radiographic progression through 2 years of guselkumab therapy were low [19]. Furthermore, safety results through up to 1 year in DISCOVER-1 and 2 years in DISCOVER-2 were generally consistent with the safety profile of guselkumab through up to 5 years in adults with psoriasis [20]. Of note, the phase 2 and DISCOVER-1 studies allowed enrollment of a limited number of patients who had previously received one or up to two, respectively, prior TNFi. However, not all TNFi-experienced patients were classified as TNFi-IR, with some patients discontinuing their prior TNFi for reasons other than inadequate efficacy or intolerance (e.g., financial/insurance, patient preference, and contraindication).

Subsequently, a phase 3b study, COSMOS, evaluated the efficacy and safety of the guselkumab 100 mg Q8W dosing regimen specifically in TNFi-IR patients with PsA [21]. In COSMOS, significantly greater proportions of patients receiving guselkumab Q8W achieved improvements in clinically efficacy, physical function, and HRQoL assessments at Week 24 compared with the placebo group, with response rates maintained through 1 year of guselkumab therapy [21]. The safety results of COSMOS were consistent with the known safety profile of guselkumab in PsA; however, the study evaluated only the Q8W dosing regimen, and not Q4W, and the study duration was restricted to 1 year.

Although limited by the small number of TNFi-experienced patients (including TNFi-IR) receiving guselkumab Q4W, and the 1-year study durations, results from DISCOVER-1 [22] and COSMOS [21] suggested that more frequent dosing with guselkumab may provide incremental benefit for TNFi-IR patients in achieving stringent treatment targets such as minimal disease activity (MDA). To date, both the United States Food and Drug Administration (US FDA) and European Medicines Agency (EMA) have approved the guselkumab Q8W dosing regimen for adults with active PsA; the Q4W dosing regimen was also approved by the EMA for patients at high risk of radiographic progression [13, 23]. The phase 3b SOLSTICE study was designed to assess both guselkumab dosing regimens (100 mg Q4W and Q8W) through 2 years in patients with PsA who have experienced either inadequate efficacy or intolerance to only one prior TNFi in order to further our understanding of the efficacy and safety of guselkumab in TNFi-IR patients, who represent a sizeable population requiring alternate treatment options.

### Methods and design

**Fig. 1:**
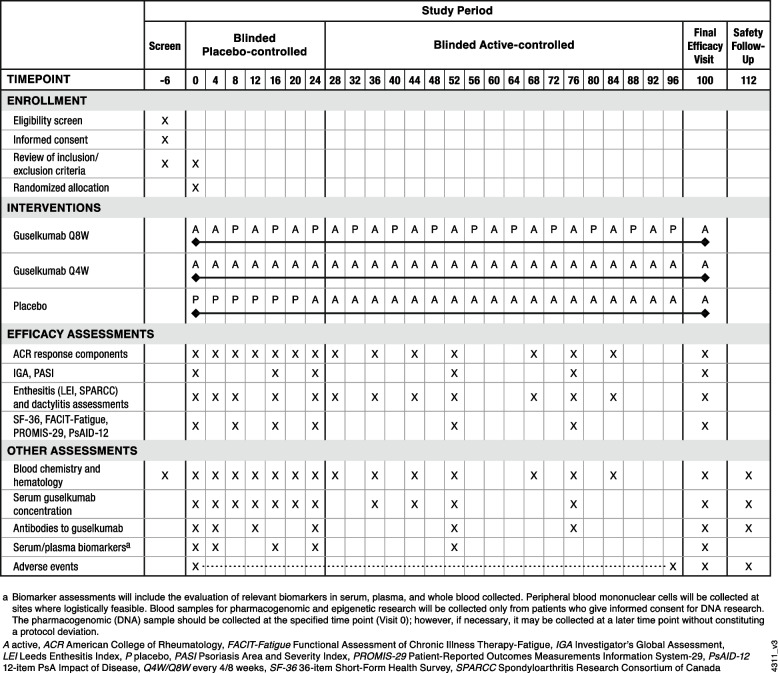
Standard protocol items: recommendation for interventional trials (SPIRIT) figure: trial visits and assessments

SOLSTICE is a phase 3b, randomized, double-blind, placebo-controlled, parallel group, multicenter (200 study centers in 13 countries), interventional study evaluating the efficacy and safety of guselkumab in TNFi-IR patients with active PsA (Fig. 1). This study follows the Standard Protocol Items: Recommendations for Interventional Trials (SPIRIT) reporting guidelines [24, 25].

### Study population and eligibility

The target study population for SOLSTICE is patients with active PsA who had an inadequate response (defined as either lack of benefit and/or intolerance) following treatment with one prior TNFi (TNFi-IR). Potential participants will be recruited through several means, including referral networks, site patient databases, posters in hospitals and waiting rooms, and other advertising efforts. Following initial screening by the study investigator, ﻿participants must satisfy all inclusion criteria, including a diagnosis of PsA for ≥ 6 months and meeting ClASsification criteria for Psoriatic ARthritis (CASPAR)[26] at screening. Participants also must have active PsA (≥ 3/66 swollen joints, ≥ 3/68 tender joints, and C-reactive protein (CRP) concentration ≥ 0.3 mg/dL) and demonstrated lack of benefit from and/or intolerance to one prior TNFi therapy. A lack of benefit to the prior TNFi is defined as inadequate improvement in joint counts, physical function, or disease activity after either ≥ 12 weeks of etanercept, adalimumab, golimumab, or certolizumab pegol therapy (or biosimilar) or ≥ 14 weeks (≥ 4 doses) of infliximab (or biosimilar) as documented by the treating physician. It should be noted that SOLSTICE eligibility requires participants to discontinue their prior TNFi ≥ 4–8 weeks prior to the first study intervention administration. Further details of this and other key inclusion and exclusion criteria are provided in Table 1.

**﻿Table 1:** Selected inclusion and exclusion criteria

Inclusion Criteria	Exclusion Criteria
Aged ≥ 18 years Diagnosis of PsA for ≥ 6 months prior to first study intervention administration and meet CASPAR criteria at screening Active PsA: ≥ 3 swollen joints, ≥ 3 tender joints, and CRP ≥ 0.3 mg/dL Inadequate efficacy and or/ intolerance to TNFi therapy: active PsA despite previous treatment with one prior TNFi Active plaque psoriasis (≥ 1 plaque of ≥ 2 cm and/or psoriatic nail changes) or documented history of psoriasis ≥ 1 of the following PsA subtypes: distal interphalangeal joint involvement, polyarticular arthritis with absence of rheumatoid nodules, asymmetric peripheral arthritis, or spondylitis with peripheral arthritis	Other inflammatory diseases (e.g., RA, AS, lupus) > 1 prior TNFi Prior TNFi within 8 weeks (infliximab, intravenous golimumab), within 6 weeks (subcutaneous golimumab, adalimumab, or certolizumab pegol), or within 4 weeks (etanercept) of first study intervention administration Previous biologic therapy other than one TNFi Previous JAK inhibitor therapy Prior therapy with systemic immunosuppressants; epidural, intra-articular, intramuscular, or intravenous corticosteroids, including adrenocorticotropic hormone; or apremilast within 4 weeks of first study intervention administration Receiving ≥ 2 csDMARDs at baseline

*AS* ankylosing spondylitis, *CASPAR* ClASsification criteria for Psoriatic Arthritis, *CRP* C-reactive protein, *csDMARD* conventional synthetic disease-modifying antirheumatic drug, *JAK* Janus kinase, *NSAID* nonsteroidal anti-inflammatory drug, *PsA* psoriatic arthritis, *RA* rheumatoid arthritis, *TNFi* tumor necrosis factor inhibitor

SOLSTICE will follow clinical trial guidelines and principles outlined by the Declaration of Helsinki and current International Conference on Harmonisation Good Clinical Practice guidelines in addition to applicable regulatory and country-specific requirements. The Institutional Review Boards or Ethics Committees at each site and local health authorities in participating countries (Argentina, Australia, Bulgaria, Czech Republic, Hungary, Israel, Malaysia, Poland, Russia, Spain, Turkey, Ukraine, and the United States, including Puerto Rico) have approved the protocol; any modifications to the protocol will be reviewed by the same organizations. The study was approved by Sterling Institutional Review Board for most sites in the United States (full list available in the Supplementary Material). Prior to participation in any study-related procedures, patients will provide written informed consent; additional consent will be collected for optional pharmacogenomic analyses.

### Objectives

The overarching aim of SOLSTICE is to evaluate the efficacy and safety of guselkumab treatment in TNFi-IR patients with active PsA. More specifically, the main study objectives are to evaluate the effects of guselkumab on the signs and symptoms of PsA, psoriasis, and patient well-being and to evaluate the safety, pharmacokinetics (PK), and immunogenicity of guselkumab in TNFi-IR patients with active PsA (Table 2).

**Table 2:** Study objectives and selected endpoints

Objectives	﻿Endpoints
**Primary**	
To evaluate the efficacy of guselkumab treatment in patients with active PsA and inadequate efficacy and/or intolerance to a prior TNFi by assessing reduction in signs and symptoms of PsA	Proportion of patients achieving an ACR20 response at Week 24
**Major Secondary**	
To evaluate the efficacy of guselkumab on additional measures of signs and symptoms of PsA, psoriasis, and patient well-being in TNFi-IR patients with active PsA	At Week 16, proportion of patients achieving: • ACR20/50 responses At Week 24, proportion of patients achieving: • IGA 0/1 response and ≥ 2-grade reduction from baseline^a^ • PASI 90 response^a^ • ACR50/70 responses At Week 24, change from baseline ﻿in: • HAQ-DI score • SF-36 PCS score • FACIT-Fatigue score Over time, the proportion of patients achieving: • MDA • VLDA
**Other Secondary**	
To evaluate the safety of guselkumab in TNFi-IR patients with active PsA	For the duration of the study, through Week 112: • Frequency and type of AEs, SAEs, AEs leading to discontinuation of study intervention, infections, and injection-site reactions • Frequency of laboratory abnormalities (chemistry, hematology) maximum toxicity (Common Terminology Criteria for Adverse Events [5.0]) grades
To evaluate the PK and immunogenicity of guselkumab in TNFi-IR patients with active PsA	Through Week 112^b^: • Mean/median serum guselkumab concentrations over time • Summary of incidence of antibodies to guselkumab

*ACR20/50/70* ≥ 20%/50%/70% improvement in American College of Rheumatology response criteria, *AE* adverse event, *FACIT-Fatigue* Functional Assessment of Chronic Illness Therapy-Fatigue, *HAQ-DI* Health Assessment Questionnaire-Disability Index, *IGA* Investigator’s Global Assessment, *MDA* minimal disease activity, *PASI 90* ≥ 90% improvement in Psoriasis Area and Severity Index, *PsA* psoriatic arthritis, *SAE* serious adverse event, *SF-36 PCS* 36-item Short Form Healthy Survey physical component summary score, *TNFi-IR* tumor necrosis factor inhibitor﻿-inadequate responder, *VLDA* very low disease activity

^a^Among patients with ≥ 3% body surface area affected with psoriasis involvement and an IGA score ≥ 2 at baseline

^b^Serum samples are to be collected at the final visit from patients who discontinue study intervention or who withdraw from the study. Samples will be collected before study intervention administration at visits when study intervention administration is scheduled

### Assessments

**Fig. 2:**
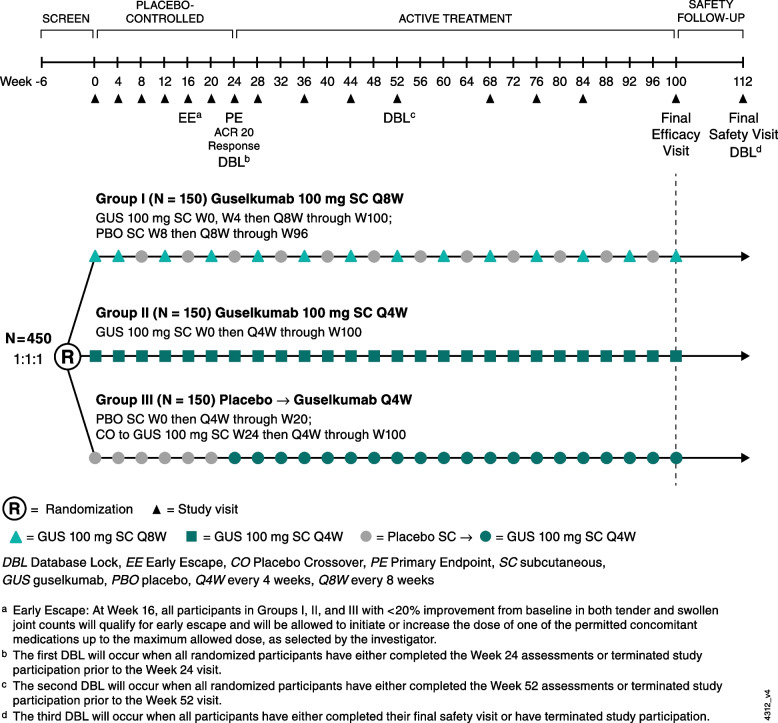
SOLSTICE study schema

The frequencies of study visits and assessments are shown in Figs. 1 and 2**.** Key efficacy assessments include swollen (0–66) and tender (0–68) joint counts, patient-reported pain (visual analog scale [VAS], 0–10), Patient Global Assessment of Disease Activity (PtGA) of arthritis (VAS, 0–10), PtGA of arthritis and psoriasis (VAS, 0–100), and Physician’s Global Assessment of Disease Activity (PhGA, VAS, 0–100). Additional psoriatic skin disease activity assessments include the Investigator’s Global Assessment of psoriasis (IGA; 0–4) [27] and Psoriasis Area and Severity Index (PASI; 0–72) [28]. Enthesitis will be evaluated utilizing the Leeds enthesitis index (LEI; 0–6) [29] and Spondyloarthritis Research Consortium of Canada (SPARCC; 0–16) methods [30]. The presence and severity of dactylitis will be assessed using a scoring system from 0 to 3 (0 – no dactylitis, 1 – mild dactylitis, 2 – moderate dactylitis, and 3 – severe dactylitis) [31]. Serum high-sensitivity CRP (mg/dL) levels, indicative of overall levels of systemic inflammation, will also be measured throughout the study. Among patients identified by the investigator as having spondylitis at enrollment, axial symptoms will be assessed using the Bath Ankylosing Spondylitis Disease Activity Index (BASDAI, 0–10), representing the average of six VAS scores, used to rate patient fatigue, spinal pain, peripheral joint pain, enthesitis, severity of morning stiffness, and the duration of morning stiffness (each from 0–10) [32].

Additional patient-reported outcome instruments include: the Health Assessment Questionnaire-Disability Index (HAQ-DI, 0–3) to assess physical function [33], the 36-item Short Form Healthy Survey physical and mental component summary scores (SF-36 PCS/MCS, 0–100) to assess general HRQoL [34], and Functional Assessment of Chronic Illness Therapy-Fatigue (FACIT-Fatigue, 0–52) to evaluate fatigue [35]. The Patient-Reported Outcomes Measurements Information System-29 questionnaires will also be utilized to assess various aspects of HRQoL: depression, anxiety, physical function, pain interference, fatigue, sleep disturbance, and ability to participate in social roles and activities [36, 37]. The 12-item PsA Impact of Disease (PsAID-12) will also be utilized in this study. This instrument assesses patient perception, rated from 0–10, of the following domains: pain, fatigue, skin, work and/or leisure activities, function, discomfort, sleep, coping, anxiety, embarrassment, social life, and depression [38]. Weighted domain scores of the PsAID-12 are combined and divided by 20 for a final total score of 0–10.

Joint evaluations (swollen and tender joints, dactylitis, enthesitis) will be performed by a designated independent joint assessor (IJA) at each study site who will only perform joint evaluations and will be blinded to all other aspects of the patient’s clinical condition. Skin assessments (IGA and PASI) will be performed by the treating physician or designee. Training will be provided by the sponsor for these joint and skin evaluations to aid in consistency across sites.

Adverse events (AEs) reported by the patient or, when appropriate, by a caregiver, surrogate, or the patient's legally acceptable representative, will be documented throughout the study, with a final safety follow-up visit at Week 112. Physical examination of vital signs, such as pulse/heart rate and blood pressure (systolic and diastolic), will be assessed at each study visit. Blood samples will be collected at regular intervals for PK and immunogenicity assessments; collected samples may also be evaluated to investigate aspects of safety or efficacy as applicable during or after the study period. Assessments may include the evaluation of relevant biomarkers in serum, plasma, and whole blood collected, to examine the biologic response to treatment and to identify markers that are relevant to guselkumab treatment and/or PsA, where local regulations permit. Blood samples for pharmacogenomic analyses will be obtained from patients who provide additional consent for pharmacogenomic testing, where local regulations permit.

### Outcomes

The primary endpoint is the proportion of patients achieving an ACR20 response at Week 24 (Table 2). An ACR20 response is defined as ≥ 20% improvement from baseline in both swollen (66 joints) and tender (68 joints) joint counts, and in at least three of the following five assessments: patient-reported pain, PtGA (arthritis), PhGA, HAQ-DI, and serum CRP level [6]; ACR50 and ACR70 responses are defined similarly using improvement thresholds of ≥ 50% and ≥ 70%, respectively. Improvements in overall disease activity will also be assessed by the proportion of patients achieving MDA/very low disease activity (VLDA) (Table 2). MDA and VLDA require meeting at least five or all seven, respectively, of the following criteria: tender joint count ≤ 1, swollen joint count ≤ 1, PASI ≤ 1, patient-reported pain VAS ≤ 15, PtGA VAS (arthritis and psoriasis) ≤ 20, HAQ-DI score ≤ 0.5, and ≤ 1 tender entheseal point [7]. Key secondary endpoints include the proportions of patients achieving ACR20 (Week 16), ACR50 (Weeks 16 and 24), and ACR70 (Week 24) responses; an IGA 0/1 response (IGA score of 0/1 and ≥ 2-grade reduction from baseline) and ≥ 90% improvement from baseline in PASI (PASI 90) at Week 24, both among patients with ≥ 3% body surface area affected by psoriasis and an IGA score ≥ 2 at baseline; and MDA/VLDA over time.

The frequency of AEs, serious AEs (SAEs), AEs leading to discontinuation of study intervention, infections, and injection-site reactions will be reported. Other AEs of special interest will include cases of newly identified malignancies or active tuberculosis.

### Study design

SOLSTICE includes a screening phase (approximately 6 weeks); a treatment phase of approximately 2 years, comprising﻿ a double-blind placebo-controlled period from Week 0 to Week 24 and an active treatment period from Week 24 to Week 100; and a safety follow-up phase extending 12 weeks after the last intended dose at Week 100 (final safety visit at week 112). Eligible patients (*N* = 450) will be randomly assigned (1:1:1) to receive subcutaneous (SC) guselkumab 100 mg Q4W; SC guselkumab 100 mg at Week 0, Week 4 and Q8W; or SC placebo with prespecified crossover to SC guselkumab 100 mg Q4W at Week 24 (placebo→guselkumab Q4W, Fig. 2). At baseline, concomitant use of NSAIDS, oral corticosteroids, and/or one csDMARD (methotrexate, sulfasalazine, hydroxychloroquine, or leflunomide) will be permitted at stable dosages. All other csDMARDs and apremilast must be discontinued ≥ 4 weeks prior to the first study drug administration. Participants who meet the early escape criteria at Week 16 (< 20% improvement from baseline in both tender and swollen joint counts) will continue to receive their randomized study treatment but will be allowed, at the investigator’s discretion, to initiate or increase the dosage of one permitted csDMARD and thus may receive a total of two csDMARDs. All other participants may initiate or increase the dosage of one additional csDMARD at the investigator’s discretion after Week 52.

### Intervention

At Weeks 0 and 4, patients will receive an SC injection (administered by study site personnel) of guselkumab 100 mg (1 mL) or matching liquid placebo for guselkumab through a single-use prefilled syringe assembled with the Ultrasafe PLUS™ Passive Needle Guard. Beginning at Week 8, patients (or caregivers) may administer the study agent after appropriate training and under supervision by site personnel, at the discretion of the investigator. From Week 32 through the end of the study, patients (or caregivers) may self-administer study injections at home with the appropriate training; however, in-person visits are required at Weeks 36, 44, 52, 68, 76, 84, 100, and 112 for prespecified efficacy and safety assessments and collection of blood samples.

Although previous studies have demonstrated the efficacy of guselkumab in adults with active PsA, a placebo control, identical in appearance to guselkumab, will be used to establish the effects of guselkumab on clinical endpoints that may occur in the absence of active intervention in this subpopulation of PsA patients. Currently available data for treating PsA TNFi-IR patients with guselkumab are limited largely to the Q8W dosing regimen and only within a 1-year time frame [21]. In the context of these limited data, the use of a placebo control is necessary in this study with the primary objective of establishing the efficacy of guselkumab for the treatment of TNFi-IR PsA patients.

Patients who discontinue the study agent will be encouraged to return for all remaining study visits through Week 24 (if discontinued prior to Week 24) or Week 100 (if discontinued after Week 24) and a final safety visit approximately 12 weeks after their final study drug administration.

### Statistical methods

Efficacy analyses at Week 24 will be conducted using the full analysis set (i.e., all randomized patients), and results will be summarized by randomized treatment group.

#### Treatment estimands

The primary endpoint, ACR20 response at Week 24, and major secondary endpoints will be analyzed based on the Adjusted Composite Estimand comprising the following components: 1) population (randomized TNFi-IR patients with active PsA); 2) treatment (placebo or guselkumab); 3) variable (ACR20 response at Week 24) wherein responders met the response criteria and had no intercurrent events [ICEs] 1–3 (Table 3) prior to that time, in the hypothetical situation where Natural Disaster or Major Disruption and associated ICE categories 4 and 5 did not occur; 4) ICEs (Table 3); and 5) population-level summary.

**Table 3:** Intercurrent events for analyses of the primary and major secondary endpoints

1	Discontinuation of study intervention for any reason other than Natural Disaster^a^ or Major Disruption^b^
2	Initiated or increased the dose from baseline of csDMARDs or oral corticosteroids for PsA
3	Initiated protocol-prohibited medications/therapies for PsA
4	Discontinuation of study intervention due to Natural Disaster^a^ or Major Disruption^b^
5	Severe treatment noncompliance (≥ 2 doses of study intervention missed) due to Natural Disaster or Major Disruption

*csDMARD* conventional synthetic disease-modifying antirheumatic drug, *PsA* psoriatic arthritis

^a^Natural Disaster defined as site closure, site access restriction, or lockdown caused by COVID-19

^b^Major Disruption defined as disruption in Ukraine and neighboring countries/territories beginning February 24, 2022

#### ICE strategy and missing data

For the primary endpoint analysis using the Adjusted Composite Estimand, the occurrence of ICEs 1–3 through Week 24 will be considered as a treatment failure (i.e., ACR20 nonresponder) regardless of ACR20 response status. Data observed following ICEs 4 and 5 will be considered as missing at random and not used. Missing data due to Natural Disaster or Major Disruption will be assumed to be missing at random and imputed using multiple imputation on the individual ACR components; missing data owing to other reasons will be imputed as no response (nonresponder imputation). Analyses of the major secondary endpoints will be conducted using the same strategy for binary endpoints, with patients considered as nonresponders if missing data for reasons other than Natural Disaster or Major Disruption. For continuous endpoints, missing data for any reason will be assumed to be missing at random and imputed using multiple imputation.

For binary endpoints, comparisons between each guselkumab group and the placebo group (difference versus placebo with 95% confidence interval [CI]) will be performed using a Cochran-Mantel–Haenszel test stratified by baseline use of csDMARDs (yes/no﻿) for each imputation set, and the Wilson-Hilferty transformation will be applied across the imputation sets. Treatment comparisons for continuous endpoints will be performed using an analysis of covariance. Subgroup analyses utilizing a logistic regression model to generate odds ratios and 95% confidence intervals will be performed to evaluate consistency of treatment effect in the primary efficacy endpoint across demographic characteristics and baseline disease characteristics and medication use.

Database locks will occur at Week 24 to compare the guselkumab groups with placebo and at Weeks 52 and 112 to examine the maintenance and trajectory of response through 1 and 2 years, respectively. No interim analyses are planned.

#### Safety

AEs will be summarized through Week 112 by actual treatment received and will include all patients who receive ≥ 1 (partial or complete) study agent administration.

### Randomization and blinding

Implementation of central randomization will minimize bias in the assignment of patients to intervention groups, increase the likelihood that baseline demographic and disease characteristics are evenly balanced across treatment groups, and enhance the validity of statistical testing between the guselkumab and placebo groups. Central randomization, balanced by using randomly permuted blocks stratified by baseline csDMARD use (yes/no), of patients to the guselkumab Q4W, guselkumab Q8W, or placebo→guselkumab Q4W group utilizes a computer-generated randomization schedule prepared before the study under the supervision of the sponsor.

It is planned to enroll a total of 450 patients (150 patients per intervention group) in SOLSTICE. This sample size is estimated to have > 90% power in detecting differences between each guselkumab group and the placebo group for the primary endpoint (ACR20 at Week 24), assuming a 2-sided alpha level of 0.05. These calculations were based on Week 24 ACR20 response rates of 59%, 52%, and 22% for the Q4W, Q8W, and placebo groups, respectively, in DISCOVER-1 [15] and 44% and 20% for the Q8W and placebo groups, respectively, in COSMOS [21]. Like SOLSTICE, these studies enrolled patients with ≥ 3 swollen and ≥ 3 tender joints; DISCOVER-1 also required participants to have a CRP level ≥ 0.3 mg/dL.

At the Week 24 database lock, the data will be unblinded to a limited number of sponsor personnel for analysis of the primary and major secondary endpoints (Table 2) while patients are still participating in the study. Identification of sponsor personnel who will have access to the unblinded patient-level data will be documented prior to unblinding. Investigative study sites and patients will remain blinded to initial treatment assignment until after the Week 112 database lock. The blind will not be broken until all patients have completed the study and the database analyses are finalized. However, in the event of a medical emergency, a process exists by which investigators can determine the identity of the intervention.

### Oversight and monitoring

A Trial Steering Committee of independent members has been created for study consultation purposes, consistent with previous studies [14–16]. Steering Committee members are responsible for advising on the strategic direction of the trial; providing clinical expertise on clinical study parameters (e.g., program design, population, endpoints); reviewing, analyzing, and interpreting the data from the trial; and providing guidance on important analyses to inform clinical practice. Steering Committee members, including employees of the sponsor, will remain blinded for the duration of the study.

Sponsor personnel will monitor study site conduct to ensure adherence to the protocol and Good Clinical Practice through central, remote, and on-site monitoring. Because guselkumab has been approved by regulatory agencies in several countries for use in adults with active PsA, an independent Data Safety Monitoring Board was not established for the SOLSTICE study.

## Discussion

Among patients with PsA receiving a TNFi, approximately 40% do not achieve an ACR20 response and a majority do not achieve MDA within 6 months of treatment initiation [5, 8]. Cycling to another TNFi does not always provide lasting control of disease activity, as evidenced by the decreased persistence observed with subsequent TNFi treatments [9, 10]. Patients who have experienced inadequate efficacy with, or intolerance to, TNFi therapies represent a difficult-to-treat patient population, and current treatment guidelines are unclear on the optimal treatment strategies for these patients.

Safety findings across seven psoriasis studies and four PsA studies utilizing data from over 4,000 patients through up to 5 years of follow-up showed that the rates/100 patient-years for all AEs and SAEs were similar between guselkumab and placebo [39]. There were no cases of Crohn’s disease or ulcerative colitis, and few *Candidiasis* infections occurred in guselkumab-treated patients, with an incidence of 0.50/100 patient-years. Among patients with PsA, the types and rates of AEs were consistent across both biologic-naïve and TNFi-experienced patients through up to 2 years [40]. Of note, current GRAPPA guidelines recommend against the use of IL-17A inhibitors in patients with comorbid IBD owing to the risk of exacerbation [41], and these therapies have also been associated with an increased risk of *Candidiasis* [42]. Additionally, the potential for an increased risk of cardiovascular events with the use of JAK inhibitors has been observed [43–45].

Evidence from the pivotal phase 3 guselkumab clinical trials demonstrated that guselkumab is efficacious in improving the signs and symptoms of PsA in both biologic-naïve and TNFi-experienced patients, regardless of dosing regimen [15–19]. While the phase 3b COSMOS study corroborated the safety and efficacy of the guselkumab Q8W dosing regimen in TNFi-IR patients, the Q4W dosing regimen was not evaluated in this specific patient population [21]. Limited data from a small cohort of TNFi-experienced patients in DISCOVER-1, including those who discontinued their prior TNFi due to inadequate efficacy, found that response rates for achieving ACR50, ACR70, IGA 0/1, and MDA were numerically higher in patients receiving guselkumab Q4W than in those receiving the Q8W dosing regimen [15, 17, 22].

Thus, SOLSTICE, a phase 3b, multicenter, randomized clinical trial, will allow for a more comprehensive evaluation of the efficacy and safety of selectively inhibiting the IL-23p19 subunit with guselkumab in PsA patients who have experienced either inadequate efficacy with and/or intolerance to one prior TNFi. The results from this study will provide insight as to whether TNFi-IR patients with treatment-resistant PsA may benefit, in terms of depth and durability of response, from more frequent guselkumab administration. Findings from SOLSTICE and a separate, pragmatic, randomized trial designed to compare the efficacy and safety of switching to a new mechanism of action (guselkumab) versus cycling to a second TNFi (SC golimumab) in TNFi-IR patients (EVOLUTION; clinicaltrials.gov: NCT05669833) are expected to yield valuable information to advance treatment guidelines for these patients.

In post hoc analyses of pooled data from the DISCOVER-1 and DISCOVER-2 studies, guselkumab demonstrated consistent response across several different subgroups of PsA patients [46]. Given that the SOLSTICE study will provide longer-term efficacy and safety data in TNFi-IR patients through up to 2 years and includes both the Q4W and Q8W dosing regimens, findings are expected to provide additional insight into the benefit-risk assessment of both dose regimens in this particular PsA subpopulation. Of note, the SOLSTICE study findings will be augmented by real-world data on the effectiveness of guselkumab in PsA patients who have been previously treated with multiple TNFi and/or other biologic DMARDs in the CorEvitas patient registry [47]. Furthermore, a separate phase 3b study, APEX, has been designed to gather additional data on the effects of both guselkumab Q4W and Q8W dosing regimens on structural damage in a PsA population enriched for patients with risk factors for future radiographic progression [48].

SOLSTICE will assess both guselkumab 100 mg Q4W and Q8W dosing regimens in the largest phase 3 study population of PsA patients to-date and will enroll only TNFi-IR patients. This study population will be enriched with patients who are inadequate responders to only one prior TNFi and are otherwise biologic-naïve. Findings from SOLSTICE will provide critical information on the efficacy and safety of selectively inhibiting the IL-23p19 subunit in TNFi-IR PsA patients that will inform treatment decisions in this distinct patient population.

## Trial status

The first patient was screened on September 28, 2021, and the final study visit is expected to be completed on September 7, 2026. Protocol Amendment 1, 12 May 2022.

### Supplementary Information

**Supplementary Material 1.**

## Data Availability

Not applicable.

## Abbreviations

ACR: American College of Rheumatology
AE: Adverse events﻿
AS: Ankylosing spondylitis﻿
BASDAI: Bath Ankylosing Spondylitis Disease Activity Index
CASPAR: ClASsification criteria for Psoriatic Arthritis
CI: Confidence interval
CRP: C-reactive protein
csDMARD: Conventional synthetic disease-modifying antirheumatic drug
FACIT-Fatigue: Functional Assessment of Chronic Illness Therapy-﻿Fatigue
HAQ-DI: Health Assessment Questionnaire-Disability Index
HRQoL: Health-related quality of life
ICE: Intercurrent event
IGA: Investigator’s Global Assessment
IJA: Independent joint assessor
IL: Interleukin
IR: Inadequate responder
JAK: Janus kinase
LEI: Leeds Enthesitis Index
MDA: Minimal disease activity
NSAID: Nonsteroidal anti-inflammatory drug
PASI: Psoriasis Area and Severity Index
PhGA: Physician’s Global Assessment of Disease Activity
PK: Pharmacokinetics
PsA: Psoriatic Arthritis
PsAID-12: 12-Item PsA Impact of Disease
PtGA: Patient’s Global Assessment of Disease Activity
Q4W: Every 4 weeks
Q8W: Every 8 weeks
RA: Rheumatoid arthritis﻿
SAE: Serious adverse event
SC: Subcutaneous
SD: Standard deviation
SF-36 MCS/PCS: 36-Item Short Form Health Survey Mental/Physical Component Summary Score
SPARCC: Spondyloarthritis Research Consortium of Canada
SPIRIT: Standard Protocol Items: Recommendations for Interventional Trials
TNFi: Tumor necrosis factor inhibitor
VAS: Visual analog scale
VLDA: Very low disease activity
