# Supplementary material for: Efficacy and safety of guselkumab in patients with active psoriatic arthritis who had inadequate efficacy and/or intolerance to one prior tumor necrosis factor inhibitor: study protocol for SOLSTICE, a phase 3B, multicenter, randomized, double-blind, placebo-controlled study
Source: BMC Rheumatol. 2024 May 21;8:20. doi: 10.1186/s41927-024-00386-7 (PMC11106968; doi:10.1186/s41927-024-00386-7)
Supplement: Supplementary file 1 — Supplementary Material 1. [file 41927_2024_386_MOESM1_ESM.docx]

| **Supplemental Table 1. List of Institutional Review Boards/Ethics Committees in SOLSTICE** | |
| --- | --- |
| **Country** | **Institutional Review Board/Ethics Committee** |
| **Argentina** | Comité de Ética en Investigación Clínica (Barclay) Parana 775 6 piso A y B Ciudad Autonoma de Buenos Aires 1071 |
|  | CIREC Comite institucional de revision de ensayos clinicos Av Luis Maria Campos  726 Ciudad Autonoma de Buenos Aires Buenos Aires C1426BOR |
| **Australia** | Bellberry Human Research Ethics Committee 123 Glen Osmond Road Eastwood South Australia 5063 |
|  | Monash Health Human Research Ethics Committees 246 Clayton Rd Clayton Victoria 3168 |
|  | Western Health EC Office for Research Level 3 Western Centre for Health Research & Education (CHRE) Sunshine Hospital Furlong Road St Albans Victoria 3021 |
|  | South West Sydney Local Health District Research and Ethics Office Level 2 UNSW Clinical School Liverpool Hospital Liverpool New South Wales 2170 |
|  | Eastern Health Office of Research & Ethics 5 Arnold St. Box Hill Victoria 3128 |
| **Bulgaria** | Ethics Committee for Clinical Trials 8 Damian Gruev Str. Sofia 1303 |
| **Czech Republic** | Eticka komise pro multicentricke klinicke hodnoceni Fakultni nemocnice v Motole V Uvalu 84 Praha 5 150 06 |
|  | Eticka komise Revmatologicky ustav Na Slupi 4  Praha 2 128 50 |
| **Hungary** | Medical Research Council Ethics Committee for Clinical Pharmacology Széchenyi István tér 7-8. Budapest 1051 |
| Israel | Helsinki Committee - Meir Medical Center 59 Tchernekovski St. Kfar Saba 44281 |
|  | Helsinki Committee Chaim Sheba Medical Center Derech Sheba 2 Tel-Hashomer 5265601 |
|  | Helsinki Committee - Bnai-Zion Medical Center 47 Golomb St Haifa 31048 |
|  | Helsinki Committee - Carmel Medical Center 7 Michal St Haifa 3436212 |
|  | Helsinki Committee of Barzilai Medical Center 2 Ha-Histadrut St Ashkelon 78278 |
|  | Helsinki committee - Rambam MC Rambam Medical Center 8 Haaliya Hashniya St Bat Galim Haifa 31096 |
| **Malaysia** | Medical Research And Ethics Committee (MREC) Block A Kompleks Institut Kesihatan Negara (NIH) No 1 Jalan Setia Murni U13/52  Seksyen U13 Bandar Setia Alam Shah Alam Selangor 40170 |
| **Poland** | Komisja Bioetyczna przy Dolnośląskiej Izbie Lekarskiej ul. Kazimierza Wielkiego 45  Wrocław  50-077 |
| **Russia** | Independent Interdisciplinary Committee for Ethical Expertise of Clinical Trials 51 Leningradsky Prospect Moscow 125468 |
|  | LEC of LLC Semeynaya poliklinika #4 33 Stantsionnaya St Moscow region Korolev 141060 |
|  | Local Independent Ethics Committee of the Chelyabinsk Regional Clinical Hospital 70-8 Vorovskogo St Chelyabinck 454076 |
|  | Local ethics committee at SAHI of Yaroslavl region 'Clinical Hospital #3' 61 Mayakovskogo St Yaroslavl 150007 |
|  | Intercollegiate Ethic Committee 20/1 Delegatskaya St Moscow 127473 |
|  | Ethics Committee at Medical Centre Revma-med pr. Molodezhnyy  d. 6  pom. 4 Kemerovo 650070 |
|  | Ethics Committee at FSBE Institution of Higher Education Kemerovo Sate Medical University 22A Voroshilova St Kemerovo 650056 |
|  | LEC at Saratov Regional Clinical Hospital Smirnovskoe Gorge  1 Saratov 410053 |
|  | LEC of Tula Regional Clinical Dermatovenerological Dispensary 1A M. Raskovoy 1st proezd Tula 300053 |
|  | Local Ethics Committee of Krasnoyarsk State Medical University n.a. Voyno-Yasenetskiy 1 Partizana Zheleznyaka St Krasnoyarsk 660022 |
|  | Local Independent Ethics Committee of State Budgetary Healthcare Institution The Rostov State Medical University 29 Nahichevanskiy pereulok Rostov on Don 344022 |
|  | LEC at Regional Clinical Dermatovenerological Dispensary 10c6  4th Verhniy Mikhailovsky proezd NA Moscow NA 115419 |
|  | LEC of State Budgetary Healthcare Institution of the Samara Region "City clinical hospital #5 of Tolyatti" 25  Zdorovya bulvar Samara region Tolyatti 445039 |
|  | LEC at Clinical-Diagnostic Center Euromedservice  JSC 10c6 4th Verhniy Mikhailovsky proezd Moscow 129110 |
|  | Independent Ethics Committee of State Budgetary Healthcare Institution of the Moscow Region Moscow Regional Scientific Research Clinical Institute n.a. M.F. Vladimirskiy 61/2 Shchepkina ul. Moscow 129110 |
|  | Ethics Committee of Clinical Rheumatology Hospital #25 30A Bolshaya Podyacheskaya St Saint-Petersburg 190068 |
|  | LEC at FSEI Altay State Medical University 40 Lenina St Barnaul 656038 |
|  | Federal State Budgetary Educational Institution of Higher Professional Education Kirov State Medical University Ministry of Health Care of Russia Local Ethics Committee 163 Moskovskaya St. Kirov 610048 |
|  | The Local Ethics Committee of Omsk Regional Clinical Hospital 3  Berezovaya St  Omsk 644111 |
|  | Local ethic committee of State Budgetary Healthcare Institution of Nizhny Novgorod region ''Nizhny Novgorod regional Clinical Hospital n.a. Semashko'' Rodionova Str 190 build. 5 Nizhny Novgorod 603126 |
|  | Ethic Committee of JSC Family Medicine Center  1c3 Nachdiva Vasilyeva str.  Ekaterinburg  620043 |
|  | LEC at Yaroslavl Regional Clinical Hospital 7 Yakovlevskaya St Yaroslavl 150062 |
|  | Committee on Ethic of Federal State Budget Educational Institution of High Education "Privolzhsky Research Medical University" 18/1  Verkhnevolzhskaya nab. Nizhny Novgorod 603155 |
|  | Independent Interdisciplinary Committee for Ethical Expertise of Clinical Trials 51 Leningradsky Prospect Moscow 125468 |
|  | Ethics Committee of OOO Scientific-Research Center Eco-safety Yuriy Gagarin prospekt  65 Saint-Petersburg 196143 |
|  | Local IRB/IEC - Institution Details:  Independent Interdisciplinary Committee for Ethical Expertise of Clinical Trials 51 Leningradsky Prospect  Moscow 125468 |
| **Spain** | CEIC Area 7 - Hospital Clinico San Carlos de Madrid C/ Doctor Martin Lagos  s/n - Ciudad Universitaria Madrid 28040 |
| **Turkey** | Marmara University Medical of Faculty Clinical Research Ethics Commitee Başıbüyük Mah. Maltepe Başıbüyük Yolu Sok. No: 9/2 Istanbul Maltepe 34854 |
| **Ukraine** | Ethics Commission of Lviv Regional Clinical Hospital 4 Nekrasova St Lviv 79010 |
|  | Ethics Commission of SI''National Scientific Center''The M.Strazhesko Institute of Cardiology of NAMSU'' 5 Narodnogo Opolcheniya St Kyiv 03151 |
|  | Local IRB/IEC - Institution Details:  Ethics Commission of Medical Center LLC ''Health Clinic'' 1/166 Striletska Str. Vinnytsya 21009 |
|  | Local IRB/IEC - Institution Details:  Ethics Commission of Kyiv City Clinical Hospital #3 26 Petra Zaporozhtsya St Kyiv 02000 |
|  | Local IRB/IEC - Institution Details:  Ethics Commission of 'OK' Clinic 121 Kharkivske roadway Kyiv 2091 |
|  | Local IRB/IEC - Institution Details:  Ethics Commission of Kyiv Regional Clinical Hospital 1 Baggoutivska str. Kyiv 04107 |
|  | Local IRB/IEC - Institution Details:  Ethics Commission of Kyiv Railway Station Clinical Hospital #2 9 Povitroflotsky av. Kyiv 3049 |
|  | Ethic Commission of Municipal Institution "Cherkasy Regional Hospital of Cherkasy Regional Council" 3 Mindeleyeva St Cherkasy 18009 |
|  | Ethics Commission of Medical Center 'Consylium Medical' 17 Hlybochytska St Kyiv 4050 |
|  | Ethics Commission of City Clinical Hospital No. 2 of Dnipropetrovsk Regional Council 2 30 years Victory St Kryvyi Rih 50000 |
|  | Ethics Commission of Communal Institution Kherson City Clinical Hospital of E.E. Karabelesh 22/1 Ushakova St Kherson 73000 |
|  | Ethics Commission of Odessa Regional Clinical Hospital 26 Zabolotnogo St Odessa 65025 |
|  | Ethics Commission of Institute of Rheumatology 25 Dniprovska Naberezhna St Kyiv 2081 |
|  | Ethics Commission of Institute of Rheumatology 25 Dniprovska Naberezhna St Kyiv 2081 |
|  | Ethics Commission of Medical Center LLC Harmony of Beauty 12 Viacheslava Chornovola St Kyiv 1135 |
|  | Ethics Commission of Municipal Institution of Sumy Regional Council “Sumy Regional Clinical Hospital” 18 Kovpaka St Sumy 40031 |
|  | Ethics Commission of Medical Center LLC 'Modern Clinic' Centralniy 20 office 210 Zaporizhzhia 69005 |
|  | Ethics Commission of MI of Tepnopil Regional Council ''Ternopil University Hospital'' 1 Klinichna St Ternopil 46002 |
|  | Ethics Commission of Vinnitsa regional clinical hospital named after M.I. Pyrogov 46 Pyrogova Str Vinnitsa 21018 |
|  | Ethics Commission of Vinnitsa regional clinical hospital named after M.I. Pyrogov  46 Pyrogova Str  Vinnitsa  21018 |
|  | Ethics Commission of Zaporizhzhya Clinical Hospital #6 34 Stalevariv str. Zaporizhzhya 69000 |
|  | Ethics Committee of 'Volyn regional clinical hospital of Volyn regional council' 21 Prezydenta Hrushevskoho Ave Lutsk 43005 |
|  | Local Ethics Committee in Poltava Regional Clinical Hospital 23 Shevchenka St Poltava 36024 |
|  | Ethics Commission of Communal nonprofit enterprise of Kharkіv Regional Council "Regional Clinical Hospital" 13 Nezalezhnosti Ave Kharkiv 61058 |
|  | Ethics Commission of Transcarpathian Regional Clinical Hospital named after Andrii Novak 22 Peremogy Str. Uzhgorod 88000 |
|  | Ethics Commission of Khmelnitckiy regional hospital 1 Pilotskaya St Khmelnitskiy 29000 |
|  | Ethics Commission of Ivano-Frankivsk City Clinical Hospital 114 Mazepy St Ivano-Frankivsk 76018 |
|  | Municipal Institution Of Health Care Kharkiv City Policlinic # 24 153 Poltavskiy shliakh St Therapeutic Department  Kharkiv 61064 |
|  | Municipal Institution Of Health Care Kharkiv City Policlinic # 24 153 Poltavskiy shliakh St  Therapeutic Department Kharkiv 61064 |
|  | Ethics Commission of LLC Medical House 'Odrex' 69/71 Rozkydailivska St Odessa 65026 |
| **United States of America** | Sterling Institutional Review Board 6300 Powers Ferry Road Suite 600-351 Atlanta, GA 30339 |
|  | University of Michigan Medical School IRB (IRBMED) 2800 Plymouth Road Building 520  Room 3214 Ann Arbor, MI 48109 |
|  | Johns Hopkins Medicine Institutional Review Board East Baltimore Campus (Central Office)1620 McElderry St. Reed Hall- B130 Baltimore, MD 21205-1911 |
|  | Southern Arizona VA Health Care System (SAVAHCS) IRB 3601 S 6th Avenue  Tucson, AZ 85723 |
